# Bloom and bust: intestinal microbiota dynamics in response to hospital exposures and *Clostridium difficile* colonization or infection

**DOI:** 10.1186/s40168-016-0156-3

**Published:** 2016-03-14

**Authors:** Caroline Vincent, Mark A. Miller, Thaddeus J. Edens, Sudeep Mehrotra, Ken Dewar, Amee R. Manges

**Affiliations:** Department of Microbiology and Immunology, McGill University, Montréal, Québec Canada; Génome Québec Innovation Centre, McGill University, Montréal, Québec Canada; Jewish General Hospital, Montréal, Québec Canada; Devil’s Staircase Consulting, North Vancouver, British Columbia Canada; New York Genome Center, New York, NY USA; Department of Human Genetics, McGill University, Montréal, Québec Canada; School of Population and Public Health, University of British Columbia, Vancouver, British Columbia Canada

**Keywords:** *Clostridium difficile* infection, Whole metagenome shotgun sequencing, Intestinal microbiota, Antimicrobials, Medications

## Abstract

**Background:**

*Clostridium difficile* infection (CDI) is the leading infectious cause of nosocomial diarrhea. Hospitalized patients are at increased risk of developing CDI because they are exposed to *C. difficile* spores through contact with the hospital environment and often receive antibiotics and other medications that can disrupt the integrity of the indigenous intestinal microbiota and impair colonization resistance. Using whole metagenome shotgun sequencing, we examined the diversity and composition of the fecal microbiota in a prospective cohort study of 98 hospitalized patients.

**Results:**

Four patients had asymptomatic *C. difficile* colonization, and four patients developed CDI. We observed dramatic shifts in the structure of the gut microbiota during hospitalization. In contrast to CDI cases, asymptomatic patients exhibited elevated relative abundance of potentially protective bacterial taxa in their gut at the onset of *C. difficile* colonization. Use of laxatives was associated with significant reductions in the relative abundance of *Clostridium* and *Eubacterium*; species within these genera have previously been shown to enhance resistance to CDI via the production of secondary bile acids. Cephalosporin and fluoroquinolone exposure decreased the frequency of Clostridiales Family XI Incertae Sedis, a bacterial family that has been previously associated with decreased CDI risk.

**Conclusions:**

This study underscores the detrimental impact of antibiotics as well as other medications, particularly laxatives, on the intestinal microbiota and suggests that co-colonization with key bacterial taxa may prevent *C. difficile* overgrowth or the transition from asymptomatic *C. difficile* colonization to CDI.

**Electronic supplementary material:**

The online version of this article (doi:10.1186/s40168-016-0156-3) contains supplementary material, which is available to authorized users.

## Background

*Clostridium difficile* infection (CDI) is the leading cause of infectious diarrhea in hospitalized patients. In the USA alone, there are an estimated 453,000 cases and 29,300 deaths from CDI each year [1]. CDI is associated with a wide range of syndromes, from asymptomatic colonization to mild diarrhea or more severe pseudomembranous colitis that may progress to toxic megacolon, intestinal perforation, sepsis, and death [2]. Despite advances in infection control practices and the development of new treatment options, there has been a steady increase in the incidence and severity of CDI in the last decade and outbreaks continue to occur in hospitals and health-care institutions worldwide [3, 4].

Hospitalized patients are at increased risk of developing CDI because they are exposed to *C. difficile* spores through contact with the hospital environment and often receive broad-spectrum antimicrobials that disrupt the integrity of the indigenous intestinal microbiota and impair colonization resistance (i.e., the ability of the microbiota to prevent the establishment of enteropathogens like *C. difficile* in the gut). Nearly all classes of antibiotics have been associated with CDI, but clindamycin, penicillins, cephalosporins, and fluoroquinolones seem to pose the greatest risk [5–7]. Additional risk factors for CDI include advanced age, underlying diseases, gastrointestinal surgery, nasogastric tube feeding, and use of proton pump inhibitors (PPIs, a class of medications that inhibit the production of gastric acid in the stomach) [2, 8].

Among patients who acquire *C. difficile* in their gut, some will remain asymptomatically colonized while others may go on to develop diarrhea or more severe forms of CDI. Differences in pathogen or host factors like the immune status or the integrity of the intestinal microbiota may affect the clinical presentation of CDI. In hospitals and health-care facilities, asymptomatic carriers often outnumber symptomatic patients and may represent a considerable reservoir of *C. difficile* that contributes to environmental contamination and disease transmission among patients [9, 10]. It has been suggested that patients with asymptomatic *C. difficile* colonization are at decreased risk of developing CDI, but a recent meta-analysis has suggested this may not be the case [11, 12]. Previously, we showed that patients who have higher levels of Clostridiales Family XI Incertae Sedis were at a decreased risk of developing CDI [13], and others have demonstrated that the presence of secondary bile acid-producing bacteria such as *Clostridium scindens* was associated with resistance to CDI [14].

Despite the strong relationship between the intestinal microbiota and CDI susceptibility, the impact of non-antimicrobial medications on the microbiota has not been examined in detail.

In this study, we prospectively examined the intestinal microbiota of hospitalized patients at-risk for CDI. Using whole metagenome shotgun (WMGS) DNA sequencing, we specifically assessed (i) the changes in the relative abundance of microbial taxa in patients who were identified as colonized or infected with *C. difficile* and (ii) the impact of antibiotics and other medications on the diversity and composition of the intestinal microbiota among patients who were neither colonized nor infected with *C. difficile*. We postulated that protective microbiota members which are thought to mediate competitive inhibition against *C. difficile* (such as Clostridiales Family XI Incertae Sedis and non-toxigenic *C. difficile*) [13, 15] or limit the germination and growth of *C. difficile* via the production of secondary bile acids (*Clostridium* and *Eubacterium* genera) [14, 16] are present in colonized but not in infected patients. We also hypothesized that not only antibiotics but also other medications such as PPIs will decrease the overall diversity of the intestinal microbiota and increase the relative abundance of opportunistic microorganisms such as enterococci and yeasts [17–20]. We report that the relative abundance of Clostridiales Family XI Incertae Sedis, *Clostridium*, and *Eubacterium* is higher in asymptomatically colonized patients than in CDI cases. Moreover, antibiotics and other medications such as laxatives have substantial effects on the intestinal microbiota of hospitalized patients and reduce the relative abundance of these potentially protective bacterial taxa. Even though the term “infected” is sometimes used to designate people who are asymptomatically colonized with *C. difficile*, in this report, we will use the term “infected” (or CDI) to describe patients who are symptomatic and “colonized” to describe patients who are asymptomatically colonized with *C. difficile*.

## Results

### Subject characteristics

A total of 104 patients were enrolled in the study. Four patients did not provide any stool sample, and six stool samples were excluded because of poor library quality or sequencing results; this left a total of 98 patients and 229 fecal samples in the study. We analyzed a median of two fecal samples per patient (range, 1–15). Patient characteristics are shown in Table 1. The majority of patients (63 %) suffered from osteoarthritis and were admitted for orthopedic surgery; most of them (89 %) received cefazolin (a first-generation cephalosporin) as perioperative antimicrobial prophylaxis. One patient (1.0 %) had asymptomatic *C. difficile* colonization on admission, three patients (3.1 %) became asymptomatically colonized during hospitalization, and four patients (4.1 %) developed hospital-acquired CDI. All of the CDI cases had diarrhea during their initial episode but none of them developed recurrent CDI.

**Table 1:** Characteristics of the study patients

Variable	Neither *C. difficile* infection nor colonization (*n* = 90)	*C. difficile* infection (*n* = 4)	*C. difficile* colonization (*n* = 4)
Age, mean years (range)	74 (61–91)	71 (66–80)	74 (71–78)
Male sex	44 (49)	2 (50)	2 (50)
Horn’s index^a^, median (range)	1 (1–2)	1 (1–1)	1 (1–2)
Duration of hospitalization^b^, median days (range)	5 (1–69)	21 (2–61)	5 (1–15)
Hospitalization in past 12 months^c^	11 (12)	0	0
Fecal specimens analyzed, median (range)	2 (1–12)	4 (1–15)	2 (1–4)
Reason for hospital admission			
Osteoarthritis/Rheumatoid arthritis	62 (69)	0	1 (25)
Pneumonia	8 (9)	1 (25)	1 (25)
Cellulitis	4 (4)	1 (25)	1 (25)
Fever	2 (2)	0	0
Chronic obstructive pulmonary disease	3 (3)	0	0
Others^d^	11 (12)	2 (50)	1 (25)
Medication use^b^			
Non-steroidal anti-inflammatory drugs	33 (37)	4 (100)	2 (50)
Proton pump inhibitors	26 (29)	2 (50)	2 (50)
Glucocorticoids	12 (13)	2 (50)	1 (25)
Opioids	25 (28)	3 (75)	1 (25)
Laxatives	16 (18)	3 (75)	0
Propulsive agents	3 (3)	0	0
Antipropulsive agents	1 (1)	1 (25)	0
Chemotherapeutic agents	0	0	1 (25)
Any antibiotic	80 (89)	4 (100)	4 (100)
Cephalosporins	60 (67)	1 (25)	1 (25)
Fluoroquinolones	13 (14)	2 (50)	0
Penicillin with β-lactamase inhibitors	12 (13)	3 (75)	1 (25)
Vancomycin (intravenous)	7 (8)	2 (50)	0
Carbapenems	6 (7)	2 (50)	2 (50)
Penicillins	5 (6)	0	0
Azithromycin	4 (4)	1 (25)	0
Metronidazole	3 (3)	1 (25)	0
Cotrimoxazole	2 (2)	0	0
Others^e^	2 (2)	3 (75)	0

Data are number (%) of subjects unless otherwise specified

^a^Evaluated at study enrollment

^b^From admission until diagnosis of *C. difficile* infection or colonization (for infected and colonized patients, respectively) or until discharge (for patients with neither infection nor colonization)

^c^Information about prior hospitalization was unknown for one of the 90 patients with neither *C. difficile* infection nor colonization

^d^Other reasons include bladder/kidney/urinary tract infection, closed fracture, urosepsis, cholecystitis, chronic stasis dermatitis, perinephric infection, diverticulitis, abdominal pain, abdominal hernia, ureteral stone, gangrene, and hip pain

^e^Other antimicrobial agents include oral vancomycin, clindamycin, daptomycin, gentamicin, Tigecycline, antivirals, and antifungals

### WMGS sequencing

Fecal samples were evaluated by WMGS sequencing to assess the diversity and composition of the intestinal microbiota. We obtained a median of 6.1 million high-quality reads per sample (range, 0.6–41.9 million). The proportion of human DNA reads was highly variable (range, 0.1–94.9 %) but half of the samples contained <20 % of human reads. After removing the host DNA, we computed the frequency of 16S ribosomal RNA (rRNA) genes in each sample as a measure of bacterial DNA content. The mean frequency of 16S rRNA genes per megabase (Mb) of sequence data was 0.39 (standard deviation 0.18). Across all samples, we obtained a median of 2.1 % of reads (range, 0.1–4.8 %) with a hit to MetaPhlAn2’s taxonomic marker database, with at least 2700 hits per sample. *Bacteroides* (99.0 %), *Prevotella* (98.0 %), and *Subdoligranulum* (98.0 %) were the most common genera, and Porphyromonadaceae (100 %), Bacteroidaceae (99.0 %), Enterobacteriaceae (98.0 %), Prevotellaceae (98.0 %), and Ruminococcaceae (98.0 %) were the most common families detected across patients (percentage of patients in which the corresponding taxa was detected).

### *C. difficile* detection

Results from the detection of *C. difficile* or its toxins in eight patients who developed *C. difficile* colonization (*n* = 4) or infection (*n* = 4) are provided in Table 2. Out of these eight patients, six had a positive *C. difficile* culture result (two of the four CDI cases did not have *C. difficile* detected by toxigenic culture but rather with an enzyme immunoassay). All patients with a positive *C. difficile* culture result had a single isolate recovered and characterized. Two of the four asymptomatic carriers were colonized with a toxigenic strain. *C. difficile* was also detected by WMGS sequencing in two of the four asymptomatically colonized patients as well as in two CDI patients with a positive *C. difficile* culture result. No other patients had *C. difficile* detected by culture or sequencing.

**Table 2:** Detection of *C. difficile* and its toxins in four asymptomatically colonized and four CDI patients

Patient ID	Sample type	Toxigenic culture^a^		Enzyme immunoassay^c^	WMGS sequencing	
		Culture	Toxin^b^		No. of reads	Relative abundance of *C. difficile* (%)
Asymptomatically colonized						
30	Rectal swab	Positive	Negative	ND	8,487,324	0.000
63	Rectal swab	Positive	Positive	ND	10,366,360	0.000
87	Rectal swab	Positive	Negative	ND	4,489,956	0.363
99	Stool	Positive	Positive	ND	14,952,432	0.064
CDI						
35	Stool^d^	Negative	ND	Positive	5,926,081	0.000
36	Stool^d^	Positive	Positive	ND	7,068,998	0.017
55	Stool	Positive	Positive	Positive	9,950,687	0.003
98	Stool	Negative	ND	Positive	4,337,018	0.000

*WMGS* whole-metagenome shotgun, *ND* not done

^a^Performed by Dale N. Gerding’s laboratory

^b^Detected by restriction endonuclease analysis or cytotoxicity assay

^c^Performed on a stool sample at the Jewish General Hospital

^d^A rectal swab was used for WMGS sequencing

### Intestinal microbiota dynamics in patients with *C. difficile* colonization or infection

**Fig. 1:**
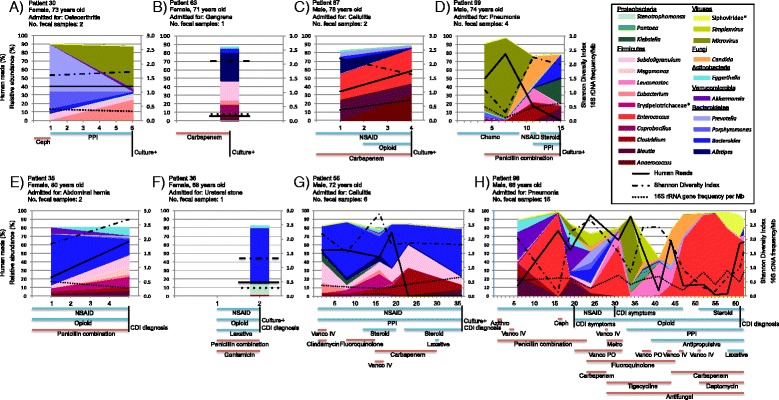
Intestinal microbiota dynamics in patients with *C. difficile* colonization or infection. The figure shows changes in intestinal microbiota composition (*area charts* or *bar charts* for patients with a single measurement), Shannon diversity (*dash-dotted line*), bacterial DNA content (*dotted line*), and human DNA content (*solid line*) during hospitalization for patients who developed *C. difficile* colonization (**a**–**d**) or infection (**e**–**h**). Only those microbial taxa with a relative abundance of ≥10 % in at least one sample are depicted. The *y*-axis on the left shows the relative abundance of microbial taxa or human DNA proportions, while the *y*-axis on the right shows the Shannon diversity index or bacterial DNA content (expressed as number of 16S rRNA genes per Mb). The *x*-axis shows the number of days after hospital admission (day 0). The figures only display information for the period from hospital admission until the last stool collection. The *bars underneath the graphs* indicate hospital exposures: *Azithro* azithromycin, *Ceph* cephalosporin, *Chemo* chemotherapeutic agent, *Metro* metronidazole, *NSAID* nonsteroidal anti-inflammatory drug, *Penicillin combination* penicillin with β-lactamase inhibitor, *PPI* proton pump inhibitor, *Steroid*, glucocorticoid, *Vanco IV* intravenous vancomycin, *Vanco PO* oral vancomycin. The day on which a patient had a positive *C. difficile* culture (Culture+), the presence of *C. difficile* infection symptoms, but a negative *C. difficile* enzyme immunoassay (CDI symptoms) or a diagnosis of *C. difficile* infection (CDI diagnosis) is also indicated

Figure 1 shows variations in the relative abundance of microbial taxa, overall microbial diversity, as well as bacterial and human DNA proportions in relation to hospital exposures and length of stay for patients who became colonized (Fig. 1a–d) or infected (Fig. 1e–h) with *C. difficile*. The human DNA content and the composition and diversity of the intestinal microbiota were highly variable during patient hospitalization. In contrast, the proportion of bacterial DNA (measured after the exclusion of human DNA) was more stable.

We specifically looked at the relative abundance of potentially protective bacterial taxa, including Clostridiales Family XI Incertae Sedis, *Clostridium*, and *Eubacterium*. A few days prior to or at the time of *C. difficile* colonization, three out of four asymptomatic patients exhibited high relative abundance of these taxa in their intestinal microbiota: the relative abundance of *Eubacterium* was 24.3 % in patient 30 on the day *C. difficile* colonization was detected, the relative abundance of *Anaerococcus* (a Clostridiales Family XI Incertae Sedis member) was 31.3 % in patient 87 on the day *C. difficile* colonization was detected, and the relative abundance of *Clostridium* spp. other than *C. difficile* (mainly *Clostridium bolteae*) was 22.2 % in patient 99 4 days prior to *C. difficile* colonization. We also observed transient increases in the relative abundance of *Clostridium* spp. other than *C. difficile* (31.0 % [mainly *C. bolteae and Clostridium hathewayi*] 13 days prior to CDI diagnosis, patient 55) or Clostridiales Family XI Incertae Sedis (17.4 % 37 days prior to CDI diagnosis, patient 98; data not shown) in two of the four patients who developed CDI. However, these increases occurred several days prior to CDI onset and the corresponding bacterial taxa had a relative abundance of less than 5 % upon CDI diagnosis. In the fecal samples (*n* = 196) from the 90 patients who were neither colonized nor infected with *C. difficile*, the median abundance (range) of Clostridiales Family XI Incertae Sedis, *Clostridium*, and *Eubacterium* was 3.1 (0.0–62.3 %), 0.4 (0.0–67.6 %), and 0.2 % (0.0–37.0 %), respectively.

We observed transient but large increases of *Microvirus* (reaching a relative abundance of ≥50 % in patients 30, 98, and 99), *Candida* (reaching a relative abundance of >30 % in patients 98 and 99), and *Leuconostoc* (reaching a relative abundance of >15 % in patients 98 and 99). In patients 98 and 99, the increase in the relative abundance of *Microvirus* was concomitant with an increase of human read proportions and a decrease of microbial diversity. In patient 98, striking blooms of *Enterococcus* occurred on days 16 (98.0 %), 31 (60.8 %), and 47–62 (64.8–97.5 %) after admission and appeared to follow the administration of intravenous vancomycin. In the fecal samples (*n* = 196) from the 90 patients who were neither colonized nor infected with *C. difficile*, the median abundance (range) of *Microvirus*, *Candida*, *Leuconostoc*, and *Enterococcus* was 0.0 (0.0–95.6 %), 0.0 (0.0–3.5 %), 0.0 (0.0–51.0 %), and 0.1 % (0.0–91.7 %), respectively.

### Medication use and intestinal microbiota diversity and composition

We examined the effect of antibiotics as well as other medications on the diversity and composition of the gut microbiota. To avoid confounding the results, we excluded patients who were colonized or infected with *C. difficile* (*n* = 8) from these analyses; therefore, the *Clostridium* genera does not include *C. difficile*. Utilization of fluoroquinolone was moderately correlated with the use of metronidazole (*ρ* = 0.45), as were use of penicillin with use of gentamicin (*ρ* = 0.57), and use of opioids with use of laxatives (*ρ* = 0.41).

Among patients who were neither infected nor colonized with *C. difficile*, we obtained a mean Shannon diversity index of 2.0 (standard deviation 0.6). Exposure to fluoroquinolones and intravenous vancomycin was associated with a significant decrease in intestinal microbiota diversity, while use of opioids was associated with an increase in diversity (Table 3).

**Table 3:** Associations between medication use and intestinal microbiota diversity

Medication^a^	No. of patients exposed	No. of samples exposed	Estimate^b^	Standard error	*P* value^c^
Antibiotics					
Cephalosporins	57	81	−0.06	0.07	0.3764
Fluoroquinolones	8	15	−0.20	0.10	*0.0367*
Penicillin with β-lactamase inhibitors	7	12	−0.17	0.19	0.3805
Carbapenems	5	11	−0.30	0.26	0.2417
Intravenous vancomycin	6	12	−0.41	0.15	*0.0080*
Other medications					
Glucocorticoids	7	13	−0.02	0.17	0.8977
Laxatives	13	24	−0.05	0.09	0.5976
Non-steroidal anti-inflammatory drugs	30	57	0.08	0.08	0.3264
Opioids	23	38	0.13	0.06	*0.0202*
Proton pump inhibitors	21	54	−0.11	0.10	0.2738

Among patients who were neither colonized nor infected with *C. difficile*

^a^Received within 3 days prior to stool collection

^b^A positive value indicates that intestinal microbiota diversity was higher among patients exposed to the medication, while a negative value indicates that intestinal microbiota diversity was lower

^c^ *P* values were determined by using the GEE-derived robust *z* scores

A large number of microbial taxa were affected by common hospital exposures; out of a total of 125 genera and 59 families identified in at least 5 % of the patients, 88 genera (70.4 %) and 42 families (71.2 %) were associated with a statistically significant increase or decrease in relative abundance in univariable analyses (Additional file 1: Table S1 and Additional file 2: Table S2). With the exception of non-steroidal anti-inflammatory drugs (NSAIDs) and opioids, most medications tended to decrease, rather than increase, the relative abundance of affected microbial taxa. Carbapenems (33 genera and 13 families) and intravenous vancomycin (34 genera and 15 families) influenced the largest number of microbial taxa.

In multivariable analyses, we specifically looked at the impact of medications on the relative abundance of microbial taxa that are thought to be protective against CDI (Clostridiales Family XI Incertae Sedis, *Clostridium*, and *Eubacterium*) as well as opportunistic microorganisms that can overgrow as a result of antimicrobial use (*Enterococcus* and *Candida*) (Table 4). Use of laxatives was associated with significant reductions in the relative abundance of *Clostridium* and *Eubacterium*. Administration of cephalosporins, fluoroquinolones, or penicillin with β-lactamase inhibitor was associated with significant reductions in the frequency of Clostridiales Family XI Incertae Sedis. Exposure to fluoroquinolones significantly increased the relative abundance of *Enterococcus*, while intravenous vancomycin was of borderline significance. None of the medications were associated with significant variations in the relative abundance of *Candida* in multivariable analyses.

**Table 4:** Multivariable analysis of medications associated with selected microbial taxa

Taxa (outcome)	Medication^a^	Estimate^b^	Standard error	*P* value^c^
*Clostridium*	Glucocorticoids	3.72	1.65	0.0239
	Laxatives	−2.46	0.99	0.0126
	Non-steroidal anti-inflammatory drugs	−2.11	0.90	0.0197
*Eubacterium*	Fluoroquinolones	−0.99	0.44	0.0237
	Laxatives	−1.21	0.48	0.0115
Clostridiales Family XI Incertae Sedis	Cephalosporins	−5.78	2.08	0.0055
	Fluoroquinolones	−5.95	2.21	0.0071
	Non-steroidal anti-inflammatory drugs	3.76	1.99	0.0580
	Penicillin with β-lactamase inhibitors	−5.70	2.54	0.0248
*Enterococcus*	Fluoroquinolones	215.43	102.34	0.0353
	Intravenous vancomycin	181.32	95.02	0.0564

Among patients who were neither colonized nor infected with *C. difficile*. All of the multivariable models were adjusted for age, sex, and duration of hospitalization

^a^Received within 3 days prior to stool collection

^b^A positive value indicates that the relative abundance of the corresponding taxa was higher among patients exposed to the medication, while a negative value indicates that the relative abundance was lower

^c^ *P* values were determined by using the GEE-derived robust *z* scores

## Discussion

We previously demonstrated that a reduction in Clostridiales Family XI Incertae Sedis is significantly and independently associated with the risk of CDI in a distinct patient population [13]. Certain species within the *Eubacterium* and *Clostridium* genera, notably *C. scindens*, have the ability to convert primary bile acids into secondary bile acids [16] and have been shown to strongly inhibit *C. difficile* in the intestinal microbiota of antibiotic-treated mice and humans [14]. In this study, asymptomatically colonized patients, but not CDI cases, exhibited elevated relative abundance of Clostridiales Family XI Incertae Sedis, *Clostridium*, or *Eubacterium* in their gut shortly prior to or when *C. difficile* colonization was detected. These observations are in agreement with our initial hypothesis and suggest that co-colonization with any of these potentially protective bacterial taxa might prevent *C. difficile* overgrowth in colonized subjects or the transition from asymptomatic colonization to a full-blown infection.

Previous studies have shown that in the absence of gross perturbation, the intestinal microbiota of healthy subjects is relatively stable over time [21, 22]. In contrast, we observed dramatic shifts in the composition and diversity of the intestinal microbiota in patients who developed *C. difficile* colonization or infection, as well as in other hospitalized patients (data not shown). These observations suggest that single measurements of the intestinal microbiota may be problematic when studying hospitalized patients, in particular, as their microbiota composition is strongly influenced by their illness and medical interventions. This offers one explanation why some studies examining the role of the intestinal microbiota on health outcomes may yield conflicting results.

In a previous study, we showed that fecal excretion of human DNA was significantly increased in patients with CDI or having other gastrointestinal problems and appeared to be an outcome of intestinal inflammation [23]. In this study, the proportion of human DNA was highly variable over time but was observed to be higher prior to CDI development in two of the four cases. In some of our patients, increases in the proportion of human DNA were concomitant with a reduction of microbial diversity and increases in the relative abundance of *Microvirus*. Microviruses are single-stranded DNA bacteriophages that infect enterobacteria and are commonly found in human gut samples [24]. High levels of human DNA excretion have been associated with a reduction of intestinal microbiota diversity [23].

One of the patients who developed CDI (patient 98) exhibited striking blooms of *Enterococcus* (with frequencies reaching 98 %) on multiple occasions during his hospitalization. These blooms appeared to follow the administration of intravenous vancomycin and may represent an overgrowth of vancomycin-resistant *Enterococcus* (VRE). Studies have shown that the use of intravenous vancomycin is significantly associated with VRE colonization or infection [25]. Although we do not know whether this patient actually carried VRE or developed sepsis, Ubeda et al. have previously shown that intestinal domination by VRE typically precedes bloodstream infection in hospitalized patients [26]. In the cohort of patients who were neither colonized nor infected with *C. difficile*, we confirm that the use of intravenous vancomycin is associated with increases in the relative abundance of *Enterococcus*.

Receipt of opioids was associated with significant increases in microbial diversity. This medication typically delays gastrointestinal transit time and may facilitate microbial growth in the colon, thereby increasing diversity. Significant reductions in intestinal microbiota diversity were only associated with the use of fluoroquinolones and intravenous vancomycin. Surprisingly, exposure to other antibiotics and PPIs did not reduce overall microbiota diversity in our cohort but did influence the relative abundance of numerous microbial taxa. The association between PPIs and CDI development is still a controversial issue [27]. The notion of reduced microbiota diversity may suggest that the number and relative abundance of microbial taxa are both declining. However, our study suggests that microbiota dynamics are much more complex; many taxa are being depleted while others are blooming. Therefore, measurement of the overall diversity is masking important distortions in the composition of the microbial community.

In multivariable analyses, we showed that the use of laxatives is associated with significant reductions in the relative abundance of *Clostridium* and *Eubacterium*; certain species among these genera may confer protection against CDI via the production of secondary bile acids. However, in a post hoc analysis, the Kyoto Encyclopedia of Genes and Genomes (KEGG) pathway corresponding to secondary bile acid biosynthesis (ko00121) was not detected in any our metagenomic samples. Laxatives accelerate gastrointestinal transit time and may have detrimental effects on the intestinal microbiota. van der Wulp et al. previously showed that treatment with polyethylene glycol (an osmotic laxative) changes the composition of the intestinal microbiota and decreases the production of secondary bile acids in rats [28]. We also demonstrated that the use of cephalosporins, fluoroquinolones, and penicillin with β-lactamase inhibitors is significantly and independently associated with reductions in the relative abundance of Clostridiales Family XI Incertae Sedis representatives. Since cephalosporin and fluoroquinolone exposure, as well as depletions of Clostridiales Family XI Incertae Sedis, have been associated with CDI risk [13], this provides a potential explanation for why these antibiotics increase the susceptibility to CDI in hospitalized patients.

We observed discrepancies in the detection of *C. difficile* or its toxins by culture, WMGS sequencing, and enzyme immunoassay. In two of the four asymptomatically colonized patients, *C. difficile* was detected by culture but not by WMGS sequencing. In two of the four CDI cases, *C. difficile* could not be detected, either by culture or sequencing (their CDI diagnosis was based on a positive enzyme immunoassay performed at the hospital). This may be due to a low organism load, the lack of biomass collected with the rectal swabs, or insufficient sequencing depth. One patient (patient 98) received vancomycin and metronidazole treatment for presumptive CDI prior to the actual diagnosis by enzyme immunoassay, which may have reduced the quantity of *C. difficile* organisms available for detection.

The number of patients who developed *C. difficile* colonization or infection was limited in our study; therefore, we could not perform statistical analyses to assess the role of hospital exposures and microbial taxa for patients with these outcomes. In all of the four patients who developed asymptomatic colonization, *C. difficile* was detected by culture in the last stool collected prior to patient discharge. Therefore, we could not determine whether *C. difficile* colonization was transient (present in only one occasion) or not in these patients. In the full cohort, insufficient numbers of patients exposed to certain medications (e.g., metronidazole and cotrimoxazole) or colonized by specific microbial taxa (e.g., *Aerococcus* and Microbacteriaceae) precluded statistical analyses. Diet and other factors such as underlying disease state can affect the intestinal microbiota and could still confound the associations identified in our study.

## Conclusions

The integrity of the intestinal microbiota is intricately related to the health of the host. In this study, we show that the diversity and composition of the intestinal microbiota in hospitalized patients is highly dynamic. Use of antibiotics, as well as other non-antimicrobial medications, particularly laxatives, has a profound and rapid effect on the structure of the intestinal microbiota and significantly decreased the relative abundance of key bacterial taxa that may be involved in CDI protection. Our results also support the hypothesis that co-colonization with key bacterial taxa such as Clostridiales Family XI Incertae Sedis, *Clostridium*, or *Eubacterium* may prevent *C. difficile* overgrowth or the transition from asymptomatic *C. difficile* colonization to CDI. A better understanding of CDI pathogenesis, including the medical exposures that undermine the effectiveness of colonization resistance and the specific microbiota alterations that allow *C. difficile* to infect the gut, will contribute to our ability to develop novel clinical strategies to prevent or treat this life-threatening infection.

## Methods

### Patient recruitment and follow-up

Research subjects were recruited as part of a prospective cohort study conducted at the Jewish General Hospital (JGH) in Montréal between October 2009 and April 2011. Patients ≥60 years of age, who were expected to stay more than 2 days in the hospital at the time of study enrolment, and who had received antimicrobials in the previous 48 h or were expected to receive some in the next 24 h were eligible to participate in the study. Patients admitted to the hospital for CDI were excluded. Subjects were enrolled in the study within 5 days after admission to selected medical wards or surgical units. Patients were followed for 60 days after study enrolment or 30 days after hospital discharge (whichever came first) to ascertain the development of CDI. CDI cases were followed for 60 days after successfully completing CDI treatment to monitor the development of recurrent CDI.

### Ethics, consent, and permissions

All study participants provided informed written consent. The Research Ethics Boards of the JGH (08-118A) and the McGill University Health Centre (11-205 GEN) approved the research protocol.

### Sample and epidemiologic data collection

Fecal specimens were obtained from each patient at study enrollment, every 3 days during hospitalization, at the onset of diarrhea (if applicable), and at discharge. The samples were collected as bulk stool or with the use of a rectal swab if the patient was not producing stool. Rectal swabs are a convenient means of sampling the human gut and give highly reproducible microbiota profiles that can closely resemble that of fecal samples [29]. Epidemiologic information was extracted from the patients’ medical charts and included patient demographics, previous hospitalizations, reason of admission, disease severity index, dates of admission and discharge, CDI diagnosis, and the use of antimicrobials and other medications during hospitalization (with corresponding start and stop dates).

### Toxigenic *C. difficile* culture

Toxigenic culture test results were provided as a courtesy of Dr. Dale N. Gerding (Hines VA Hospital and Loyola University Chicago Stritch School of Medicine, IL). Briefly, stool or rectal swab specimens were streaked onto selective pre-reduced cefoxitin-cycloserine-fructose agar supplemented with taurocholate (TCCFA) and incubated anaerobically for 48 h. Presumptive identification of *C. difficile* was based on typical colony morphology. If colony morphologies suggested that a mixture of *C. difficile* strains were present, multiple colonies were picked and saved individually. All rectal swabs were also inoculated onto non-selective blood agar to detect the growth of intestinal microbiota and confirm proper sampling technique, thereby excluding the possibility of false-negative results on the TCCFA. Purified isolates were characterized by restriction endonuclease analysis (REA) to determine their toxin classification [30]. The Bartels Cytotoxicity Assay (Trinity Biotech) was performed to determine the presence of toxin B in *C. difficile* isolates with an undetermined REA group.

### Study definitions

CDI was defined as follows: (i) the presence of symptoms (diarrhea, fever, abdominal pain, or ileus) and a positive *C. difficile* enzyme immunoassay (ImmunoCard Toxins A&B, Meridian Bioscience, Inc.) or toxigenic culture, (ii) an endoscopic diagnosis of pseudomembranes, or (iii) a pathological/histological diagnosis of CDI. Diarrhea was defined as the passage of ≥3 new unformed stools within 24 h. Recurrent CDI was defined as a CDI diagnosis that occurs within 60 days after successfully completing the treatment for the initial episode of CDI. Asymptomatic *C. difficile* colonization was defined as a positive stool culture for *C. difficile* and the absence of a CDI diagnosis during follow-up. Colonization or infection was considered hospital-acquired if the diagnosis was made ≥48 h after hospital admission and during the study follow-up period.

### WMGS sequencing and data analysis

Total DNA was extracted from rectal swabs with use of the DNA IQ System (Promega) and from bulk stool aliquots with the use of the QIAamp DNA Stool Mini Kit (Qiagen). Multiplexed DNA libraries were prepared according to a previously described protocol that allows the generation of libraries from low amounts of input DNA [31]. WMGS sequencing was performed with 150-nucleotide read lengths on the Illumina HiSeq 2000 and 2500 platforms at the McGill University and Génome Québec Innovation Centre. Up to 14 samples were multiplexed in each sequencing lane. Reads derived from the human genome were identified and removed with BMTagger [32]. Additional quality filtering steps included the trimming of sequencing adapters and low-quality bases from the 3′ end of reads as well as the removal of short (<60 bases) and duplicate reads [33, 34]. We retrieved all reads containing the V1–V3 reverse primer sequence (which targets a segment of the 16S rRNA gene) [35] and their 3′ sequences in order to obtain 55-mers originating with the primer sequence. The frequency of 16S rRNA genes (55-mers) per Mb of sequence data was used as a measure of bacterial DNA content in each sample. High-quality reads were also analyzed by MetaPhlAn2 [36] in order to determine the family, genus, and species level relative abundances. Diversity was calculated at the genus level with the Shannon index. HUMAnN version 0.99 [37] was used in conjunction with RAPSearch2 [38] and the KEGG database [39] to evaluate the presence of the pathway for secondary bile acid biosynthesis (ko00121) in each metagenomic sample.

### Statistical analyses

We provide a descriptive analysis of microbiota composition for patients who were either colonized or infected by *C. difficile*; we specifically examined changes in the relative abundance of potential CDI*-*protecting microorganisms (i.e., Clostridiales Family XI Incertae Sedis, *Clostridium*, and *Eubacterium*). In order to assess the relationship between hospital exposures (e.g., antibiotics and other medications) and either overall microbiota diversity or relative abundance of microbiota members, we used generalized estimating equation (GEE) analyses with a Gaussian link. GEE takes into account the multiple samples per subject and the likelihood that the microbiota profiles are correlated over time within patients. Microbial diversity and relative abundances were modeled using a log transformation. In these analyses, we excluded patients who were colonized or infected with *C. difficile* and considered only those microbial taxa and medications that were present in or administered to at least 5 % of the patients (i.e., cephalosporins, fluoroquinolones, penicillin with β-lactamase inhibitors, carbapenems, intravenous vancomycin, glucocorticoids, laxatives, NSAIDs, opioids, and PPIs). Use of medication was treated as a binary variable with “1” indicating exposure within 1 to 3 days prior to stool collection and “0” indicating otherwise. We included exposure to intravenous vancomycin in our analyses, as evidence suggests that substantial amounts of the drug can be excreted in the bowel and may therefore affect the intestinal microbiota [40]. Multivariable analyses were used in order to take into account the correlation in the use of certain medications. In addition to the medications mentioned above, all multivariable models included adjustment for age, sex, and duration of hospitalization. Final multivariable models retained significant exposure variables at a *p* value of <0.05. A positive estimate value indicates that the corresponding exposure is associated with an increase in either overall microbiota diversity or relative abundance of the given microbial taxa. All statistical analyses were performed in R [41]; GEE was performed using the geepack package. *P* values were determined by using the GEE-derived robust *z* scores.

## Availability of supporting data

The sequence data supporting the results of this article are available in the National Center for Biotechnology Information Sequence Read Archive under accession number SRP064400 (http://www.ncbi.nlm.nih.gov/sra/SRP064400).

## Additional files

Additional file 1:
** Table S1. **Significant associations between medication use and intestinal microbiota genera (univariable analysis). (XLSX 433 KB) Additional file 2:
** Table S2.** Significant associations between medication use and intestinal microbiota families (univariable analysis). (XLSX 385 KB)

## Abbreviations

CDI: *Clostridium difficile* infection
PPI: proton pump inhibitors
WMGS: whole metagenome shotgun
TCCFA: cefoxitin-cycloserine-fructose agar supplemented with taurocholate
REA: restriction endonuclease analysis
GEE: generalized estimating equations
rRNA: ribosomal RNA
VRE: vancomycin-resistant *Enterococcus*
JGH: Jewish General Hospital
NSAID: non-steroidal anti-inflammatory drug
